# The physical microenvironment of hematopoietic stem cells and its emerging roles in engineering applications

**DOI:** 10.1186/s13287-019-1422-7

**Published:** 2019-11-19

**Authors:** Pan Zhang, Chen Zhang, Jing Li, Jiyang Han, Xiru Liu, Hui Yang

**Affiliations:** 1 School of Life Sciences, Northwestern Polytechnical University, Xi’an, 710072 Shaanxi People’s Republic of China; 2 Research Center of Special Environmental Biomechanics & Medical Engineering, Northwestern Polytechnical University, Xi’an, 710072 Shaanxi People’s Republic of China

**Keywords:** Hematopoietic stem cell, Bone marrow niche, Biophysical signal, Biomaterial, Engineering

## Abstract

Stem cells are considered the fundamental underpinnings of tissue biology. The stem cell microenvironment provides factors and elements that play significant roles in controlling the cell fate direction. The bone marrow is an important environment for functional hematopoietic stem cells in adults. Remarkable progress has been achieved in the area of hematopoietic stem cell fate modulation based on the recognition of biochemical factors provided by bone marrow niches. In this review, we focus on emerging evidence that hematopoietic stem cell fate is altered in response to a variety of microenvironmental physical cues, such as geometric properties, matrix stiffness, and mechanical forces. Based on knowledge of these biophysical cues, recent developments in harnessing hematopoietic stem cell niches ex vivo are also discussed. A comprehensive understanding of cell microenvironments helps provide mechanistic insights into pathophysiological mechanisms and underlies biomaterial-based hematopoietic stem cell engineering.

## General introduction

Hematopoietic stem cells (HSCs) are the common precursors of immune cells and all blood lineages [1]. Engraftment of bone marrow (BM) cells containing HSCs and multiple hematopoietic progenitor cells (HPCs) is effective in reconstituting the hematopoietic systems of patients with genetic, immunologic, or hematologic diseases. However, the limited number of primary functional HSCs with long-term repopulation potential in common sources such as BM, peripheral blood, or umbilical cord blood (UCB) poses a challenge to transplant outcomes [2, 3]. Culturing HSCs in vitro can be challenging. In vivo, BM is the preferred site where a group of HSPCs reside, in what are known as BM niches, which support signals regulating many important biological functions of HSCs in an extrinsic manner, including self-renewal, migration, proliferation, and multilineage capacity [4]. Recent advancement has been made in HSC ex vivo expansion based on the physicochemical characterization of these niches. In particular, the mechanobiological properties of the extracellular environment can provide biophysical signals that preserve cell states. Utilization of these signals promotes the development of biomaterial-based techniques for mimicking the corresponding niche. In this study, the special microenvironment of HSCs is described. A wide range of niche biophysical cues that have been proven responsible for maintaining HSC functions are reviewed. Moreover, we discuss the efforts and progress on culture scaffolds that have been developed for ex vivo survival of HSCs. Finally, current existing problems related to niche mimicry as well as future opportunities are discussed.

## The importance of HSCs in hematopoiesis

### Making sense of HSCs and the hematopoietic system

**Fig. 1:**
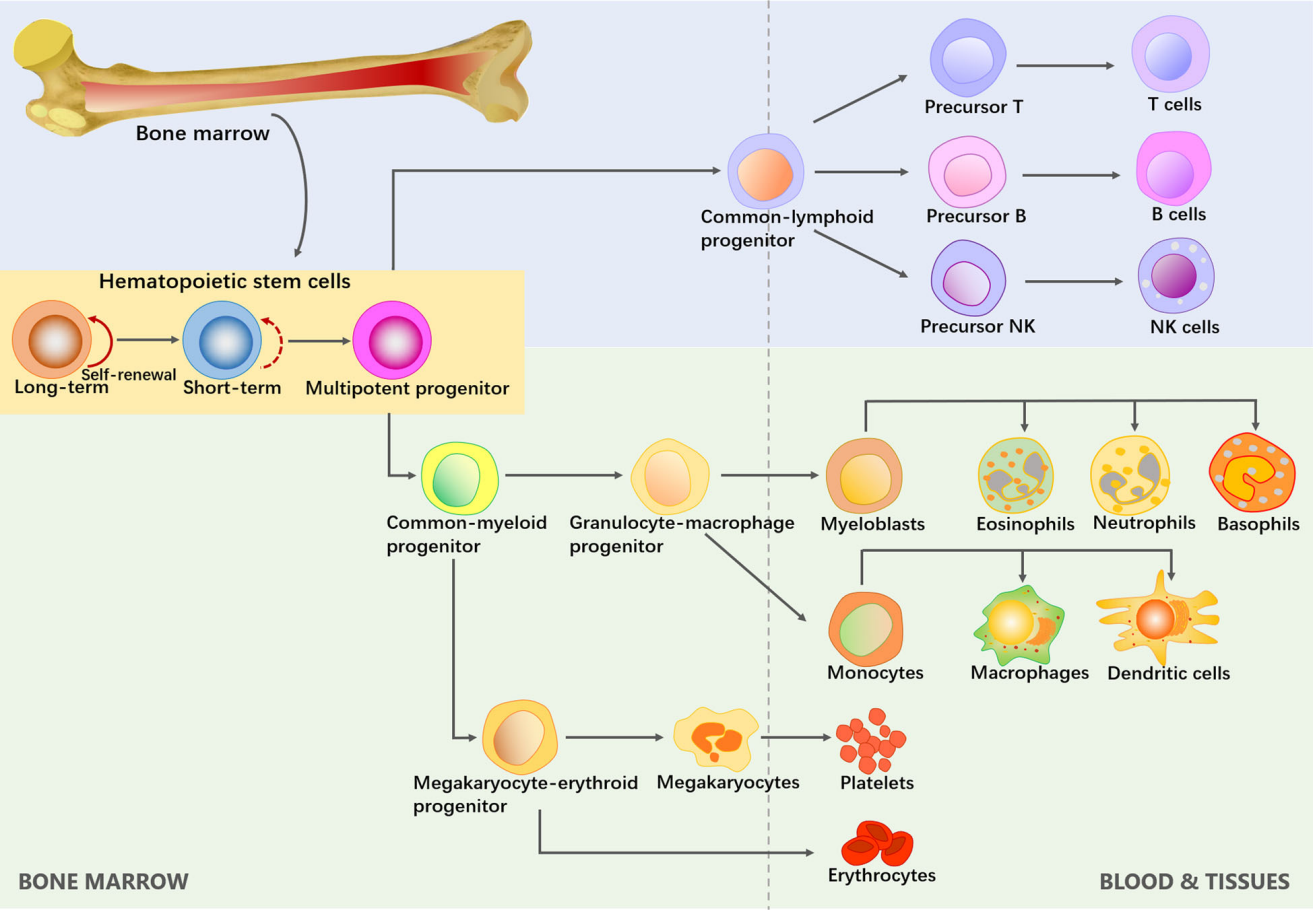
The hierarchical system model of HSC self-renewal and differentiation. HSCs locate at the top of the hematopoietic hierarchy. Multipotent progenitors have the full-lineage differentiation potential

The concept of HSCs was first proposed by Till and McCulloch. Their pioneering findings revealed the regenerative potential of single BM cells, thus establishing the existence of multipotential HSCs [5]. HSCs are the only cells within the hematopoietic system that possess the potential for both multipotency and self-renewal (Fig. 1). Multipotency is the ability to differentiate into all types of functional blood cells, while self-renewal is the ability to give rise to identical daughter HSCs without differentiation [6]. Although HSCs are defined at the single-cell level, the multipotent progenitor (MPP) pool is heterogeneous and can be divided into long-term self-renewing HSCs (LT-HSCs), transiently self-renewing HSCs (short-term HSCs, ST-HSCs), and non-self-renewing MPPs [6]. Quiescent LT-HSCs have the ability to self-renew indefinitely, mediating the homeostatic and continuous turnover of blood cells that organisms require throughout their life. ST-HSCs are generated by LT-HSCs. Highly proliferative ST-HSCs can extensively generate MPPs that have completely lost their self-renewal capacity. The downstream progenitors of ST-HSCs and MPPs ultimately produce terminally differentiated blood cells. When transplanted, however, these hematopoietic progenitors sustain hematopoiesis in the short term only and are rapidly exhausted [7].

### Clinical significance of HSCs

Mutations in hematopoietic development lead to a range of pathologies such as leukemia, myelodysplasia, and BM failure. Substantial efforts are underway to overcome the difficulties of stem cell therapy exploitation, such as transplantation and tumor purging to address various hematological disorders and malignancies [8]. HSC transplantation, which was achieved by E. Donnall Thomas in the 1950s, represents the front line of hematologic disease treatment [9]. Whole BM or HSC fractions taken from patients (autografts) or matched donors (allografts) can be infused into patients after myeloablative therapy [10]. Nevertheless, a sufficient supply is not obtainable because of the rarity of stem cells in common sources such as BM and UCB [11]. Moreover, critical hurdles remain due to the low homing efficiency of transplanted cells to the marrow cavity. Gene therapies for hematological diseases also need a robust HSC supply to offset varying degrees of inefficiency in vector-mediated transfection protocols [12]. Therefore, ex vivo expansion, which substantially increases the available cell dose, has important significance for clinical purposes. Since the culture parameters greatly influence the lineage and maturation stage of the obtained cells, HSC expansion is recognized as very challenging. Researchers have focused on the relationship between HSC biology and the microenvironment of native HSC niches. Methods to promote homing of HSCs while preserving their self-renewal capacity are emerging. Such progress offers the hope for regulating the fate of cells expanded in vitro.

## Essential microenvironment of functional HSCs

In vivo, the stem cell niche is the essential microenvironment where stem cells reside and integrate various stimuli to determine their fate. [13, 14]. Niches provide special support for cell viability. Niche-specific cell populations, extracellular matrix components, varied growth factors, and cell adhesion molecules produced by niche cells are integrated together for the common goal of controlling stem cell behavior [15]. When tissue damage occurs, niches are feedback systems for communicating information about the state of a tissue back to the related stem cells [16].

### BM HSC niches

**Fig. 2:**
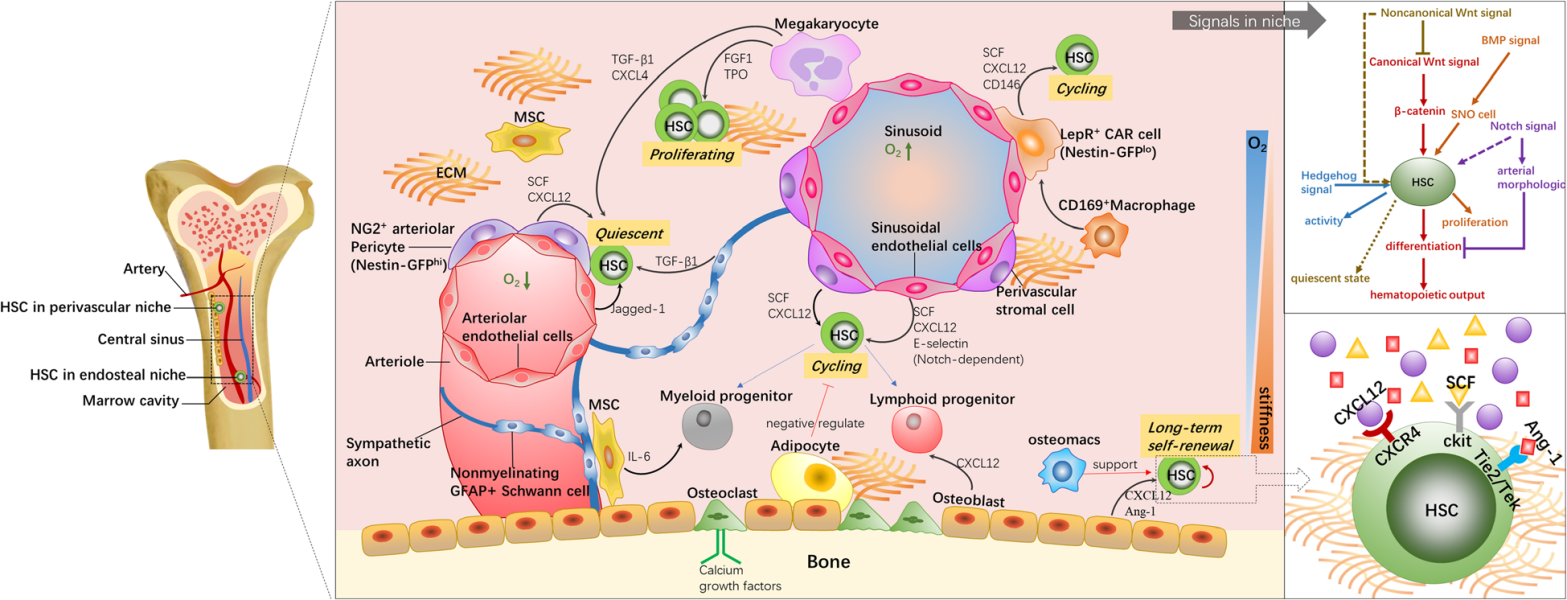
The logistic model of endosteal and perivascular niches in the bone marrow. Multiple factors such as niche cells, cytokines, signals, ECM, and oxygen concentration gradient regulate HSC activities directly or indirectly. HSCs show overall different behaviors between the different subniches. Endosteal niches contribute to the maintenance of LT-HSCs, while vascular niches activate cell cycle and initiate cell proliferation and differentiation. NG2^+^ arteriolar pericytes in arterioles subniche keep HSCs closing to arterioles in a quiescent state. LepR-expressing perisinusoidal cells are the main source of SCF and CXCL12, which are essential to HSC maintenance or mobilization

Similar to other stem cells, functional HSCs locate in unique niches. HSC niches exist in diverse tissues throughout a series of distinct embryonic developmental stages. Initial HSCs are found in the yolk sac [17]. Then, definitive hematopoiesis ensues in the aorta-gonad-mesonephros (AGM) region followed by the placenta, fetal liver, spleen, and BM [18]. Postnatally, most HSCs migrate to the trabecular regions of long bone, which offers a pivotal microenvironment for HSC quiescence, expansion, activation, and differentiation. The hematopoietic system develops along with bone formation [19]. The BM niches comprises the generally well-defined endosteal and perivascular (more specifically, arteriolar and sinusoidal) niches, which host HSCs in close proximity to osteoblasts and endothelial cells (ECs) [20]. The fates of the vasculature and bone are intertwined to create functional niches, typically including spatial and temporal variations in cellular components, the extracellular matrix (ECM), and biomolecular components [21–23] (Fig. 2). These discrete subniches exist as a series of overlapping microenvironments that communicate with each other during HSC development. Defining how these sites modulate HSC function is a crucial step toward harnessing the potential of HSCs.

### Biochemical elements regulate HSC fate

#### Niche cells and cytokines

Osteoblasts were the first cells found to support HSC expansion in vitro by presenting granulocyte colony-stimulating factor [24]. Secreted proteins such as angiopoietin-1, C-X-C motif ligand 12 (CXCL12), stem cell factor (SCF), and thrombopoietin (TPO) promote HSC growth [25–27]. Osteopontin and SDF-1α in osteoblasts are associated with HSC mobilization and egress [28]. Perivascular niche cells such as ECs, perivascular stromal cells, and mesenchymal stromal cells (MSCs) play supportive roles for physiological features of HSCs situated close to blood vessels. The heterogeneous groups of MSCs express CD146, CXCL12, nestin, and leptin receptor (LepR) for HSC survival [29, 30]. Primary ECs isolated from nonhematopoietic organs contribute HSC repopulation ability and regulate HSC pools in vitro [31]. HSC migration requires a combination of E-selectin derived from CD31^+^ ECs and ESL-1 in HSPCs. If the binding is inhibited, HSCs become relatively quiescent and resistant to irradiation [32, 33]. ECs are also found to balance the rate of proliferation and lineage-specific differentiation [28]. In addition, diversity between the arteriolar and sinusoidal niches plays disparate roles in HSC cycling. Arterial blood vessel ECs with low vascular permeability maintain HSCs in a low reactive oxygen species (ROS) environment. High-nestin-expression subsets in the arteriolar subniche keep HSCs in a dormant state [34, 35]. Sinusoidal vessels with high permeability augment HSPC activation while compromising their long-term repopulation [36]. CD146^+^ fraction and CXCL12-abundant reticular (CAR) cells secreting CXCL12, JAGGED-1, ANGPT-1, and SCF constitute essential conditional environment for HSCs situated near sinusoids [28, 37]. Different types of mature cells in BM are also recognized as important niche-modifying cells [4]. Trophic endosteal macrophages support not only osteoblast function but also the entire endosteal HSC niche. The lack of such macrophages gives rise to HSC egress into blood [38]. Nonmyelinating Schwann cells control HSC pools by activating latent transforming growth factor β [39]. Adipocytes in BM act as negative regulators for hematopoiesis. BM transplantation in mice treated with the adipogenesis inhibitor bisphenol A diglycidyl ether accelerated hematopoietic recovery compared to the results for untreated mice [40]. These various niche cells cooperate and create molecular crosstalk among HSCs and themselves, exemplifying the close relationship between angiogenesis and osteogenesis in BM [41, 42].

#### Signaling in BM niches

It has been well established that HSC fate governance depends on Notch signaling. Multiple gain-of-function studies have elucidated that Notch stimulation can be used to expand HSPCs [43]. As a direct regulator of hematopoiesis development, first, Notch signaling contributes to arterial morphological development and specification [44, 45]. Second, this signaling allows for communication between niche cells through promoting the expression of Notch receptors and ligands. The canonical Wnt pathway is another explicit environmental signaling system. Overexpression of β-catenin results in enhanced hematopoietic output [46]. Quiescent long-term HSCs express Frizzled 8, which has an antagonistic effect on Wnt signaling [47]. Wnt5a retains the quiescent state of HSCs by suppressing Wnt3a-mediated canonical Wnt signaling [48]. Indirect roles of additional signaling in HSC development have been gradually unfolded, such as BMP and hedgehog signaling. For example, BMP signaling ensures the supporting function of SNO cells for regulating the niche size and HSC fate [49]. Sonic hedgehog has been reported to promote primitive hematopoietic precursor cell proliferation and myeloid differentiation [50].

## The geometric and mechanical properties of BM niches affect HSC behavior

### Extracellular matrix of hematopoietic niches

The complex organization of the niche ECM comprises mainly macromolecules such as polysaccharide, proteoglycans, and insoluble proteins (structural protein fibers), providing a structural skeleton for niche environment. The niche can be considered a reservoir where proteins bind to proteoglycans for signal delivery [51, 52]. Scientists have recently identified how the ECM induces HSC development in vivo. Multiple ECM proteins are involved in HSC differentiation, lineage specification, proliferation, and apoptosis. For example, adhesion to fibronectin (FN) is required for long-term hematopoiesis and proliferation of human HSPCs [53]. Laminin is conducive to the homing abilities and cell cycling of HSPCs [54]. Collagen type I, mainly accumulating in the HSC endosteum niche, supports the growth of CD34^+^ HSCs [55]. It is well known that FN, collagen, and laminin have common integrin bonding domains termed RGD (Arg-Gly-Asp). RGD-binding integrins such as α4, α6, α7, α9, and β1, expressed by HSCs, interact with ECM proteins and play central roles in regulating cell development [56]. Direct ligand interactions provided by the ECM activate a variety of intracellular signaling pathways. The ECM also contributes to sequestering/releasing growth factors and morphogens in response to changes in physiological conditions, thus indirectly influencing stem cell fate [57].

### The ECM provides biophysical cues affecting HSC behavior

In addition to the biological cues mentioned above, many studies have sought to clarify the niche complexity by assessing physical cues. These cues direct multiscale developmental processes by transforming macroscale physical inputs into nanoscale molecular signals with chemical activity. Once cells sense a particular extracellular physical stimulus, complexes are formed by binding and clustering of integrins onto adhesive ligands on the substrate. Integrin-mediated signaling connects the cell actin cytoskeleton to the ECM, subsequently activating intrinsic mechanosensing pathways downstream. Similarly, biophysical signals sensed by HSCs are transmitted from integrins; however, the corresponding mechanosensing mechanism remains largely unknown and might be completely different from that of anchorage-dependent cells [58–60].

#### Influence of topography on niche cells

The ECM provides a physical scaffold for cellular constituents and supplies crucial biomechanical cues for niche supervision [61]. The ECM dimensionality and topography constitute fundamental structural features of the mechanical microenvironment that can be perceived by cells [62]. Close cell-cell interactions and three-dimensional (3D) culture conditions are often prerequisites for cell differentiation. Cells in 3D culture conditions (which are more representative of native tissues) often receive survival advantages over cells in conventional two-dimensional (2D) cultures [63]. The mechanosensitivity of BM niche cells to topographical features (e.g., nanofiber diameter, nanotube size) has gained attention. For example, MSCs cultured on a microgrooved bearing surface proliferated more rapidly than those on a 2D smooth surface, with higher expression of pluripotency-associated markers [64]. On a titanium oxide surface with relatively large nanotubes (70~100 nm diameter), MSCs exhibited 10-fold increased cell elongation and intensive selective differentiation into osteoblasts. Smaller nanotubes (~ 30 nm diameter) enhanced cell adhesion instead of accelerating cell differentiation [65]. Notably, the mechanistic basis underlying topography-mediated cell fate determination has been revealed. Briefly, cell-matrix communication induces the formation of focal adhesions. Indirect (biochemical signal-mediated) and direct (force-mediated) mechanotransduction pathways are activated to induce subsequent cell actions (adhesion, growth, migration, and self-renewal) in response to the nanotopography [66–68]. Osteoblasts and BM cells exhibited highly spread morphology and more pronounced focal adhesions on shallow (11~13 nm height) nanoislands. When the height was increased to 90 nm, this cell-spreading morphology was suppressed, and fewer stress fibers were observed [60]. In contrast, few studies have associated topographical cues with HSC fate commitment, probably because HSCs are only weakly adherent. Chua et al. first confirmed that HSPC behavior can be influenced by nanopatterning [69]. Surface-aminated polyethersulfone (PES) nanofiber meshes (529 nm diameter) enhanced cell adhesion and expansion over those of aminated films. CD34^+^/CD45^+^ cells interacted intensively with aminated nanofibers [70]. However, insufficient research has been performed to explain the specific mechanism by which HSCs accurately react to topographical features.

#### The biomechanical properties of the ECM affect HSC behavior

**Fig. 3:**
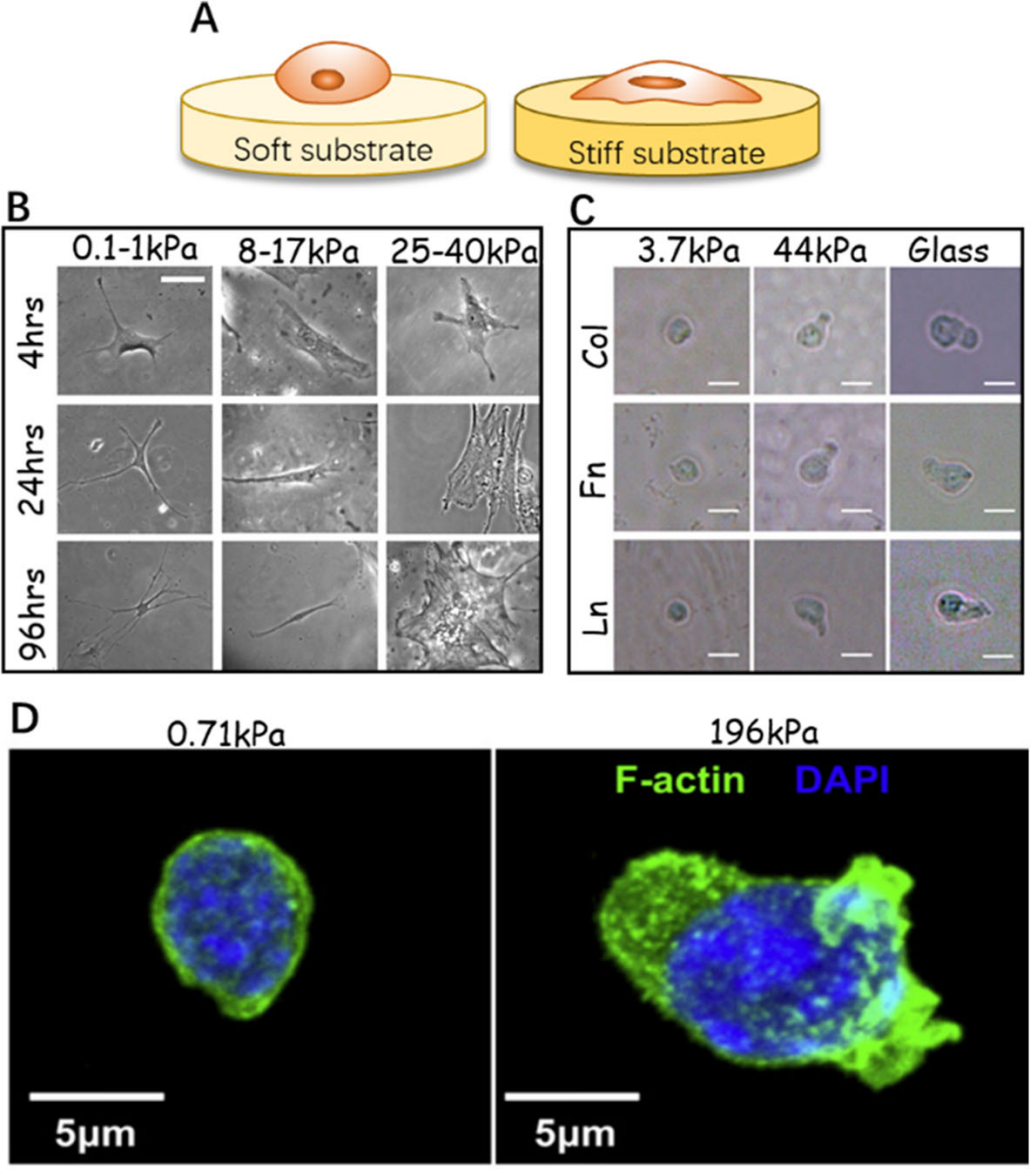
The mechanosensitivity of HSCs and MSCs in response to substrate stiffness. **a** The ECM stiffness can be mimicked by varying stiffness of the 2D substrate, which affects cell adhesion and morphology. On the stiffer substrate, cell spreads out more obviously. **b** Naive MNCs are growing on the gel surface with a range of stiffness (scale bars, 20 μm) [71]. **c** Phase-contrast images show the morphological changes of HSCs cultured on soft and hard gels. On the soft substrate, HSCs remain small and round. On the harder substrate, cell protrusions appear, accompanied by enhanced cell spreading and polarity (scale bars, 10 μm) [72]. **d** Confocal image of HSC cytoskeleton (F-actin) and nucleus (DAPI) on hard (196 kPa) and soft (0.71 kPa) matrix [73]

Given ECM diversity within tissues, biomechanical properties such as stiffness differ significantly across various HSC niches. The cellular morphology of HSCs exhibits matrix elasticity specificity (Fig. 3). The BM stiffness presents gradient variations because of its inhomogeneity. The endosteum region near the bone surface is densely populated by bone-cell progenitors and enriched with high content of FN. It is relatively stiff, with Young’s modulus of 40–50 kPa, while the stiffness of the perivascular niches surrounded by ECs as well as adipocytes is reported as approximately 3 kPa [10, 72, 74]. The central medullary region is usually an order of magnitude more compliant, at 0.3 kPa [75]. The stiffness influences HSC properties such as their terminal lineage and can be regarded as an independent factor or as a complementary factor coupled with biochemical cues. One study revealed that stronger CD34^+^ cell adhesion and faster migration were observed on stiffer (40–100 kPa) gels than on more compliant (0–20 kPa) gels in the presence of SDF-1α. The results suggested the homing preference of LT-HSCs for endosteal niches [76]. Furthermore, HSC lineage differentiation is strongly influenced by the matrix stiffness. Colony-forming unit (CFU) assays showed that CFU-E and CFU-M colonies, representing late-stage myeloid specification, increased on stiffer substrates (44 kPa), whereas softer substrates (3.7 kPa) promoted CFU-G colony formation that indicated granulocyte progenitors. Primitive CFU-GEMM colony counts, corresponding to the early stages of myeloid specification, increased on stiffer FN-coated substrates as compared to more compliant substrates. On collagen- or laminin-coated substrata, however, there was no significant change in the colony number [72]. These initial studies offer a theoretical basis for stiffness-related HSC fate control. However, it is necessary to determine whether the weak adhesion of HSCs has implications on cell mechanosensitivity to stiffness. The mechanosensing processes of obligatorily adherent cells depend on the formation of mature focal adhesions [60]. Although integrin-ECM contacts are also found in HSCs, we expect the focal adhesion forming mechanism to be distinct [59].

### Biomechanical forces regulate HSC fate

The BM stroma is a semisolid dynamic tissue. Cells residing in BM inevitably perceive a variety of physical forces, including fluid shear stress (FSS), tensile strain, hydrostatic pressure, and even mechanical unloading caused by exposure to microgravity [77]. These types of mechanical stimulation commit the regular growth of niche cells to a particular program [78]. Osteogenesis and chondrocyte maturation is directed by the cyclic biaxial tensile strain (CBTS) and cyclic hydrostatic pressure (CHB). Tensile strain loading accelerates the osteogenic differentiation of MSCs [79]. Enhanced expression of cardiac-specific marker [80] and angiogenic factors [79] were found in MSCs exposed to tensile mechanical stimuli. CHB directly influences early osteogenesis of BM-derived stem cells. During short- and long-term CHB stimulation, osteoblastogenesis of osteoblasts was temporally increased [81]. In 2018, Jung-Woog Shin et al. proposed that hydrostatic pressure and MSCs synergistically affect HSPC expansion and functional maintenance. When exposed to hydrostatic pressure, HSPCs cocultured with MSCs showed an increase in the immature HSPC phenotype and superior clonogenic potential [82]. In 2019, the same group applied intermittent hydrostatic pressure to a kind of 3D hierarchically structured scaffold to emulate dynamic BM conditions. The total cell number, CD34^+^ cell number, and cell clonogenic potential were greatly improved by the application of hydrostatic pressure [83]. This study further identified that the synergistic integration of mechanical stimuli and efficacious scaffold design offer a valuable resource for engineering microenvironments of HSCs in vitro.

Blood flow guides HSC development in the AGM region [84–86]. Blood perfusion of niches is required for the function of HSCs close to blood vessels. Low oxygen tension induced by negligible blood flow promotes enrichment of potent HSCs [87]. Generally, BM-derived stem cells undergo a range of FSS (0.86–1.51 dyn/cm) for 30–180 min per day, which maintains cell quiescence and suppresses apoptosis [88]. For osteogenesis of adipose-derived stem cells (ASCs), fluid stress induces bone mineralization and a high expression of osteogenic markers without any biochemical stimulation [89, 90]. In mouse hematopoiesis, shear stress actives signaling pathways that commit the hematopoietic potential of CD41^+^c-Kit^+^ cells [91, 92]. In one study, a suspension bioreactor was utilized for applying an agitation force at various rates, thus exposing HSCs to shear stress. The proliferation ability and differentiation of suspension-cultured HSCs were affected relative to the case of statically cultured cells. A 40-rpm rotation setting increased HSC expansion and myeloid progenitor production [93].

Hematologic abnormalities have been universally identified in humans during space flight, as mechanical loading is indispensable during the regeneration of mammalian tissues. The differentiation of hematopoietic lineages within mouse proximal femur BM can be disturbed by microgravity. Gene markers indicating early hematopoietic differentiation were down-expressed at least twofold, while bone resorption appeared on endosteal bone surfaces. Microgravity exposure facilitates hematopoietic cell differentiation toward osteoclasts, suggesting that mechanical unloading acts as an inhibitor for hematopoietic tissue formation [94]. Microgravity impairs the proliferation potential of human CD34^+^ BM cells, and the long-term repopulation potential is also degraded. However, no differences have been detected in cell apoptosis or adhesion [95, 96]. The migration potential of human CD34^+^ HSCs was reduced upon culturing in NASA’s rotating wall vessels (RWVs). Moreover, modeled microgravity promoted myeloid cell development and inhibited erythroid specificity [65]. Hence, the injury to hematopoietic cell function resulting from mechanical unloading constitutes part of the immune system damage occurring in astronauts during extended missions [96].

## Engineering HSC niches for modulating cell behavior in vitro

When engineering a synthetic HSC niche in vitro, several key criteria should be taken into account. The engineered niche should be easy to produce and scale up with the capacity for high-throughput analyses. In addition, it should afford niche elements serving as essential inducers of HSC fate and offer the possibility to examine combinations of cues [10]. Although human bone tissue can be tailored to mimetic substrates, synthetic approaches offer a more cost-effective and ready-to-use alternative. Independently controllable mechanical stimuli for modeling and manipulating the cell mechanical microenvironment can be obtained [97]. Biomaterial technology has contributed greatly to the manipulation of sophisticated surfaces/scaffolds that can provide physical (topological, spatial, and mechanical) cues, from which cells can be easily harvested via chemical or physical processes [98]. With the advancement of nanotechnology, the engineering of HSC niches has become an active area. In this section, we emphasize recent niche-mimicking approaches based on biomaterials to provide new tools to direct HSC expansion and specific lineage differentiation in vitro.

### Methods to enhance HSC-matrix interaction

Cell-matrix attachments guide how cells align themselves and ultimately promote cell multiplication or differentiation [99]. Functionalization of the material surface via peptide immobilization is one of the most widely used approaches to enhance cell adhesion. FN, laminin, and collagen types I and IV are frequently used as surface coatings [100]. An FN-coated surface supports CD34^+^ HSC differentiation into erythroids ex vivo, while laminin supports the expansion of megakaryocyte progenitors. A four-component (FN, laminin, collagen I and IV) mixed ECM coating induces cell proliferation and myeloid differentiation better than a three-component coating without collagen I [100]. The interaction of cells and ECM proteins is mediated by the nanometer-scaled lateral distance between conjugated ligands. Ligand presentation with a specific distance has been shown to regulate integrin-involved lipid raft clustering for signal complex formation. The process is influenced by the ligand type [101]. For example, small FN-derived peptide ligands supported cell adhesion at a lateral distance of less than 45 nm, while osteopontin-derived peptides enhanced cell adhesion at a larger lateral distance [102]. Peptides with a certain binding motif sometimes have higher efficiency than full-length protein. For example, the connecting segment (CS-1) motif of FN specifically binds to the membrane protein VLA4 secreted by HSCs. The expansion of CD34^+^ HSCs was significantly increased on a CS-1-immobilized PET surface, where the total nucleated cell number, total CFU, and long-term culture initiating cell (LTC-IC) number were all expanded with rates higher than those of a full-length FN-coated plate [103]. The ligand type and substrate surface nanostructure should be considered in combination when materials are evaluated. Recent studies indicate that materials secreted by niche cells also effectively build niche-mimicking surfaces and enhance cell-ECM affinity in vitro. BM-mimetic ECM scaffolds derived from MSCs were found to provide major niche proteins, osteopontin, signaling molecules, and structural cues to modulate the physical recognition process in HSC-matrix adhesion [104].

### Matrix mechanics used for niche mimicry

Tissues such as the heart, skin, bone, and vasculature have distinct mechanical properties. Multiple physiological processes, including inflammation, morphogenesis, wound healing, and cell orientation, are guided by the tissue rigidity [105]. Engler et al. first reported in 2006 that the matrix elasticity directs MSC differentiation [106]. In 2010, breakthrough research performed by Jeff Holst’s group revealed that HSCs can also be influenced by the substrate elasticity [107]. Integrated tropoelastin, which stiffens the matrix at extension lengths of > 125 nm, is required for the mechanotransduction effect on HSC maintenance. Mice Lin^−^c-kit^+^Sca1^+^ (LSK) cells cultured on an elastic tropoelastin substrate exhibited two- to threefold higher expansion than those in fresh uncultured BM. Even though cell expansion as well as the colony-forming ability was conspicuously enhanced without a cytokine supply, in the presence of cytokines, the substrate elasticity changes imparted an additive effect on this expansion. Similarly, tropoelastin increased the number of human Lin^−^CD34^+^CD38^+^ hemopoietic progenitor cells [107]. Since that work, durotaxis [105] and mechanotaxis [108] of HSCs has received strong attention. Several biomaterials have been proposed to produce hydrogels with gradients in Young’s modulus [78, 109], of which polyacrylamide (PAM) thin hydrogel layers with low elastic moduli have grown in use to mimic tissue stiffness. The gel stiffness can be changed by adjusting the composition of acrylamide/*N*,*N*-methylene bisacrylamide. Therefore, microenvironmental mechanical properties can be easily defined. Using PAM, Harley’s group demonstrated that the lineage specification of HSCs depended largely on a combinative effect of the matrix stiffness and ECM ligand type. In addition, the numbers of CFU-G and CFU-M colonies could be changed independently by altering the stiffness. Harley’s study first suggested a stiffness-mediated model for HSC early fate decision, suggesting the presence of primitive myeloid progenitors in stiffer endosteal regions and erythroid lineage specification on more compliant vascular zones [72]. PAM hydrogels showed good performance in exploring the sensitivity of HSCs to stiffness; however, the corresponding study did not estimate the influence of the changes in topography upon surface creasing, which may also determine the cell differentiation direction [110]. Possible substitutes with a smooth surface have strong application prospects.

### Three-dimensional nanofiber niche mimicry platforms

Various interwoven protein fibers compose the native ECM. Nanofibers with diameters between 50 and 300 nm mimicking the native ECM in size and structure have been successfully used for tissue engineering (TE) applications and HSC niche architecture development. Manufactured nanofibers have high stability in vivo, and their material properties are easy to optimize according to the cell type, study purpose, cell adhesion, infiltration, and porosity, which governs the transport of nutrients and oxygen [111]. On account of their mechanical, thermal, and chemical characteristics, synthetic polymers such as polyethylene terephthalate (PET), polyurethane (PU), and polyethersulfone (PES) have been employed for BM niche mimicry [112]. Although synthetic polymers intrinsically lack well-known cell-binding sites to localize signals, their exceptional flexibility in synthesis allows straightforward modification to provide such sites. In one study, the culture potency of 3D polycaprolactone (PCL) nanoscaffolds coated with FN was assessed. In comparison with the results of 2D culture, nucleated cells and CD34^+^ cells were significantly expanded. The expression of HSC markers of self-renewal increased, and the same trends were observed on homing markers. Furthermore, FN-conjugated 3D PCL scaffolds offered better clonogenicity than nonconjugated scaffolds [113]. Another method to achieve better cell adhesion is to use niche cells as a feeder layer. Better engraftment was observed in a UCB-MSC-supported scaffold compared to the results of PCL without MSCs [114, 115]. Poly(lactic-co-glycolic acid) (PLGA) can be used to produce highly porous scaffolds inlayed with pores of defined size [116]. However, PLGA meshes with an MSC layer could not support CD34^+^ cell expansion and failed to ensure overall engraftment. Potentially, the porosity, fiber size, or fiber stiffness of PLGA scaffolds is not suited for adjusting cell morphological, migratory, or adhesive properties [114]. In summary, despite their low toxicity, high cell affinity, and excellent biocompatibility, the use of synthetic polymers is relatively challenging.

Composite nanofibers such as chitosan composite, collagen composite, and gelatin composite, possessing the advantages of both natural polymers and synthetic polymers, have been fabricated for 3D cell cultivation scaffolds. The nanoscale nature of such hybrid scaffolds can be adjusted by choosing appropriate synthetic methods and seeking synergy among different types of materials [117]. For instances, PCL/gelatin fibers were investigated as promising scaffolds for cell proliferation and fate regulation [118]. PCL/gelatin ratios of 70:30 and 60:40 wt.% had good wettability and showed optimum mechanical properties for BM stromal cell growth [119]. Groups such as poly(d,l-lactide-co-glycolide)/collagen I, PCL/chitosan, and poly-d,l-lactic acid (PDLLA)/spirulina biomass have served as microenvironment mimetic scaffolds for human MSCs (hMSCs) [120], human embryonic stem cells (hESCs) [121], and breast-cancer stem-like cells [122], respectively. But whether these composite scaffolds are able to imitate the HSC niche environment for in vitro HSC culture requires further study.

### Hydrogel-based 3D biomaterials for niche mimicry

**Fig. 4:**
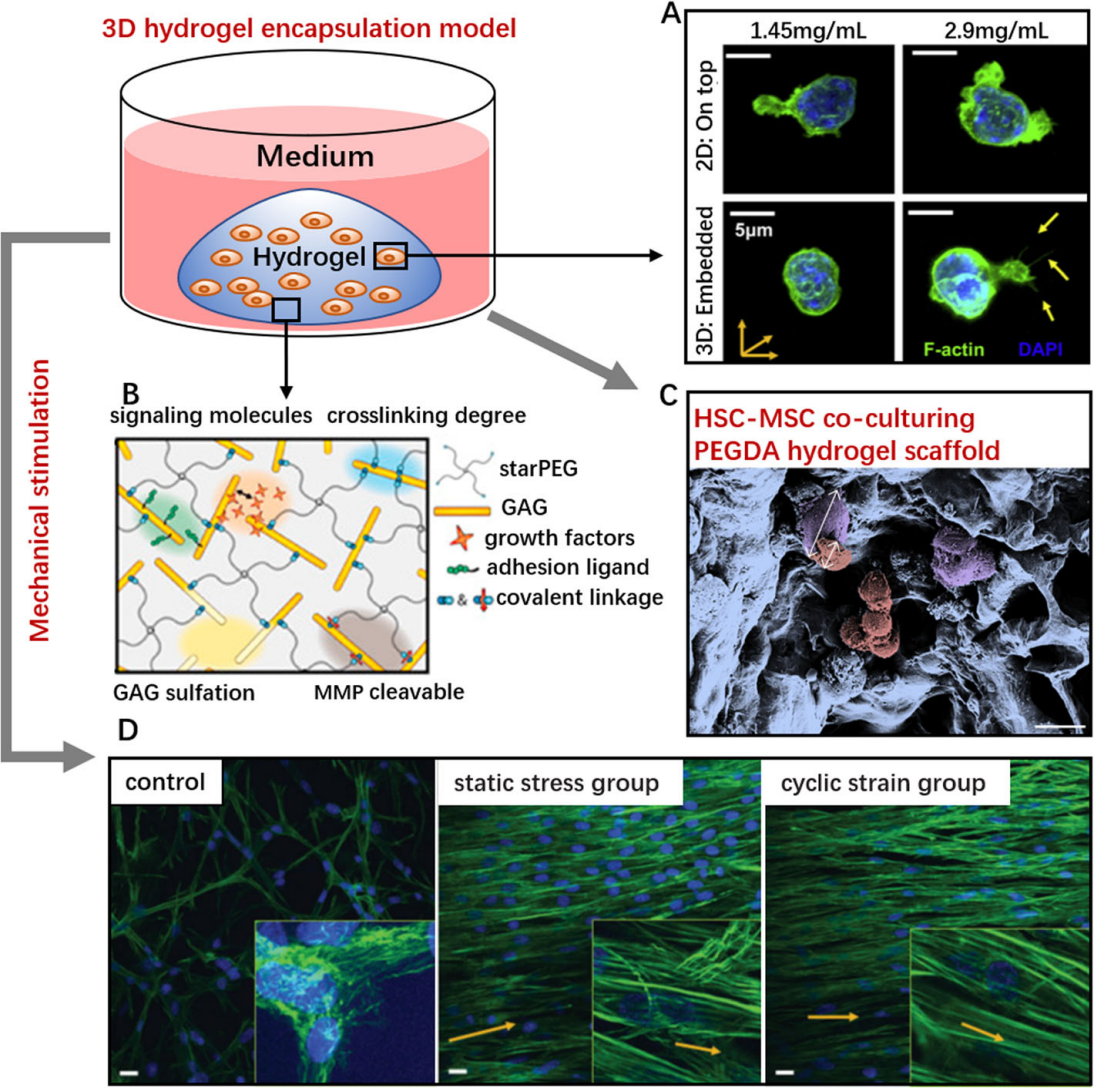
Design strategies of hydrogel encapsulation systems for HSC culture. (**a**) Confocal microscopy images of HSPC on 2D/3D collagen hydrogel constructs via cytoskeleton (F-actin) and nucleus (DAPI) staining. Yellow arrows at the lower right indicate the thin filopodial protrusions from cells are extending into the surrounding hydrogel. Scale bar, 5 μm [73]. (**b**) GAG-rich 3D starPEG-heparin hydrogel system. Gel stiffness can be controlled by the molar ratio of starPEG to heparinmaleimide [123]. (**c**) Scanning electron micrograph image of cocultured MSC-BM (purple) and HSPCs within porous PEG hydrogels. Scale bar, 20 μm [124]. (**d**) Mechanical stretching induces the morphological changes in BM-derived progenitor cells within 3D fibrin hydrogels. Stress or strain induces the organization of the surrounding matrix. Cells (nuclei, blue) and F-actin filaments (green) are randomly organized in unconstrained control group (left), while cells in the static stress group (center) and the cyclic strain group (right) are aligned parallel to the direction of the stress or strain (magnification × 40, insets × 100; scale bar,10 μm) [125]

Three-dimensional systems are representative of the actual microenvironment and can stabilize cell-environment mechanosensing and enforce the interaction between niche factors. Nanofiber platforms cannot readily mimic native spatial feature of niches because cell-environment infiltration is limited, which means that the cells lie outside rather than within the material [20]. Hydrogel encapsulated cultivation overcomes this limitation (Fig. 4). Cells can be encapsulated within complex networks with a suitable exposure area to the external environment. Therefore, encapsulation systems are well suited to represent the structural intricacies of the true microenvironment [126]. Certainly, the materials must be nontoxic to cells. After gelation, the scaffold diffusibility must ensure the exchange of nutrients and metabolites between cells and their surroundings [127]. Tunable mechanical properties, good biocompatibility, and hydrophilicity are also preferred. Natural materials that have been employed include collagen, fibrin, hyaluronic acid, alginate, and chitosan. Taking advantage of collagen I encapsulation scaffolds, Harley’s group found that the substrate dimensionality impacted HSPC viability and morphology [73]. Compared to the behavior of cells seeded on top of constructs, embedded HSPCs showed decreased cell viability but a more rounded cell shape. A stiffer 3D collagen gel guided F-actin fibers to form thin, filopodial protrusions on the edge of the cytoplasmic region (Fig. 4a). In another study, a collagen I 3D system took the lead in expounding the physical effect of the ECM matrix on cell interaction between HSCs and mature niche cells. Paracrine signals derived from Lin^+^ cells dominantly altered LSK proliferation and myeloid specification in a 2D liquid system. In a 3D environment, a low diffusivity (3 mg/ml, 1,0 HSC:Lin^+^ ratio) stabilized the HSC-generated autocrine feedback and promoted primitive progenitor fractions (LT-HSC, ST-HSC). Paracrine signals were enhanced by increasing the gel diffusivity and the ratio of HSC:Lin^+^ cells. HSC fate transitions ex vivo further indicated that the dimensionality is a critical design element for niche engineering [128].

However, natural hydrogels permit only limited controllability of the mechanical properties, and the hydrogel stiffness often varies depending on the synthesis method, gelation parameters, and type of crosslinking process [20]. The hydrogel permeability, which relies on stiffness changes, can also affect the behavior of encapsulated cells [129]. Therefore, scientists have turned to cell-embedding hydrogels composed of synthetic materials. For example, PEG hydrogel is frequently used to mimic the spongy architecture of trabecular bones. The positive effects of MSCs on HSPC proliferation within 3D PEG coculture systems were more prominent than the results of using a standard 2D platform [124]. Carsten Werner established a GAG-based starPEG-heparin biohybrid hydrogel platform [123] (Fig. 4b) that can be functionalized by a heparin fraction containing the cytokines SCF, TPO, and FLT3L; thus, biochemical and biophysical cues could be integrated ex vivo. This type of 3D environment effectively increases the LTC-IC frequency and fraction of quiescent cells (i.e., cells in G0) [123]. Furthermore, the biomechanical customization of synthetic materials enables artificial niches to offer gradient mechanical properties. This flexibility enables the materials to be used as surrogates for tissues that exhibit mechanically heterogeneity [130, 131]. In addition to the above advantages, hydrogel encapsulation is also ideal to study cell behavior as controlled by mechanical deformation (stress/strain, tension, compression, shear stress, etc.) [132, 133] (Fig. 4d).

### Platforms incorporating microfluidic technology

With the advances in materials manufacturing, numerous novel approaches have been used to construct artificial niche platforms. One promising approach is microfluidic technology. The endosteal and vascular niches heterotypically overlap in BM; microfluidic technology is capable of generating distinct compartments of cell and material cues in one biomaterial [134, 135]. Based on a microfluidic mixing platform, a kind of small-volume hydrogel containing overlapping patterns of matrix constituents was developed to study how converted signals direct cell fate. The hydrogel, which contained a gradient conjugation of SCF, increased the proliferative activity and viability of HSCs [136]. Another novel microfluidic multiorgan chip maintained UCB-HSPCs in their primitive state (CD34^+^/CD38^−^) while simultaneously retaining their differentiation capacity in the presence of MSCs [137].

## Concluding remarks and future opportunities

Signals from the microenvironment, known as the stem cell niche, enable stem cells to exhibit diverse behaviors that complement the lifelong changes. Complex niche networks are characterized by supporting cells, biomolecules generated by surrounding cells, the ECM, and major intracellular signals. Harnessing the potential of the stem cell niche forms the basis of clinical therapy. In BM, HSCs and their niches balance each other to sustain a healthy hematopoietic system. The fibrous organization of ECM components provides special topographical patterns for cell attachment. Surface pores, nanoscale features, and the topography direct cell adhesion and influence cell morphology. RGD-binding integrins allow HSCs to contact and respond accordingly to their environment. Maintaining the appropriate stiffness in BM niches is crucial for hematopoiesis and lineage specification of HSCs. A stiffness gradient between the endosteal and perivascular niches guides the behavior of different HSPC subpopulations, including motility and differentiation potency toward particular lineages. Biomechanical forces such as shear stress are pivotal for hematopoietic hierarchy development in perivascular niches. Bone unloading may be the primary origin of hematopoietic disorders in astronauts. HSCs under microgravity have impaired proliferation capacity and reduced erythroid specification capacity, reflecting the need for mechanical loading of the BM to maintain HSC functionality. Regenerative medicine seeks to imitate in vivo niche conditions to increase cell potency. As reviewed in this article, advances in fabrication approaches based on emerging biomaterials open new doors for artificial niches. Many 2D/3D engineered niches enable interaction between the environmental physical signals and HSCs. Surface modification by ECM peptides enhances cell attachment. Suitable nanometer-scaled lateral distances between ligands on the substrate surface promote HSC expansion. Varying tissue stiffness in vivo can be mimicked with 2D culture substrata produced by materials such as PAM. In recent years, the advent of 3D porous nanofiber scaffolds and hydrogel encapsulation systems has enhanced our understanding of HSC niches. Scientific evidence verifies that BM niche engineering can be inspired by obtaining insight into the physical microenvironment of HSCs. However, some critical problems remain. Present reports focus mostly on the geometric properties and rigidity of the ECM. For mimicking the dynamic microenvironment according to body activity and injury, mechanical aspects such as strain, shear stress, and hydrostatic pressure require serious consideration. Materials suitable for mechanical loading devices should be further exploited. In addition, few studies have sought to explain the detailed interaction mechanism between niche physical signals and the cell-specific physical natures of HSCs themselves. Overall, to realize high-throughput and low-volume screening for engineering applications, revolutionary approaches and additional efforts are still in demand. We anticipate that novel technologies involving biomimetic materials will overcome many of the limitations of current strategies.

## Data Availability

Data sharing is not applicable to this article as no datasets were generated during the current study.

## Abbreviations

HSC: Hematopoietic stem cell
BM: Bone marrow
HSPC: Hematopoietic stem progenitor cell
UCB: Umbilical cord blood
LT-HSC: Long-term HSC
ST-HSC: Short-term HSC
MPP: Multipotent progenitor
CMP: Common myeloid progenitor
CLP: Common lymphoid progenitor
CXCL12: C-X-C motif ligand 12
SCF: Stem cell factor
TPO: Thrombopoietin
ECs: Endothelial cells
MSCs: Mesenchymal stromal cells
ECM: Extracellular matrix
FN: Fibronectin
TE: Tissue engineering
3D: Three-dimensional
2D: Two-dimensional
